# The value of immunotherapy for survivors of stage IV non-small cell lung cancer: patient perspectives on quality of life

**DOI:** 10.1007/s11764-020-00853-3

**Published:** 2020-01-16

**Authors:** Rebekah Park, James W. Shaw, Alix Korn, Jacob McAuliffe

**Affiliations:** 1 ReD Associates, 26 Broadway Ste. 2505, New York, NY 10004 USA; 2 Worldwide Health Economics and Outcomes Research, Bristol-Myers Squibb, 3401 Princeton Pike, Lawrenceville, NJ USA

**Keywords:** Immunotherapy, Stage IV non-small cell lung cancer, Quality of life, Ethnography, Survivors, Liminality

## Abstract

**Purpose:**

The aim of this study was to examine what personally mattered to 24 patients who received immuno-oncology (IO) therapy for stage IV non-small cell lung cancer (NSCLC), as well as their families and friends, to understand how they evaluated their cancer treatments and the determinants of the quality of life (QoL) of long-term survivors.

**Methods:**

Ethnographic research was conducted with 24 patients who had responded to IO (pembrolizumab, nivolumab, atezolizumab, or durvalumab) for stage IV NSCLC, and their families and friends, evenly split among field sites in Denmark, the USA, and the UK. Data were collected using in-depth qualitative interviews, written exercises, and participant observation. Data analysis methods included interpretative phenomenological analysis, coding, and the development of grounded theory. Researchers spent 2 days with participants in their homes and accompanied them on health-related outings.

**Results:**

Our findings reveal that long-term survivors on IO experienced their journey in two phases: one in which their cancer had taken over their lives mentally, physically, and spiritually, and another in which their cancer consumed only a part of their everyday lives. Patients who survived longer than their initial prognosis existed in a limbo state in which they were able to achieve some semblance of normalcy in spite of being identified as having a terminal condition. This limbo state impacted their life priorities, decision-making, experience of patient support, and health information-seeking behaviors, all of which shaped their definitions and experience of QoL.

**Conclusions:**

The results of this study, which identify the specific challenges of living in limbo, where patients are able to reclaim a portion of their pre-cancer lives while continuing to wrestle with a terminal prognosis, may inform how cancer research can more effectively define and measure the QoL impacts of IO treatments. Also, they may identify approaches that the cancer community can use to support the needs of patients living in a limbo state. These experiences may not be adequately understood by the cancer community or captured by existing QoL measures, which were designed prior to the emergence of IO and without sufficient incorporation of contextual, patient-driven experience.

**Implications for Cancer Survivors:**

Increased awareness of the specific experiences that come with long-term survival on IO may direct how resources should be spent for cancer support for patients and their families. Expanding how QoL is evaluated based on patients’ lived experiences of IO can reflect a more accurate depiction of the treatment’s benefits and harms.

## Introduction

Lung cancer represents the leading cause of cancer-related mortality worldwide with non-small cell lung cancer (NSCLC) accounting for roughly 85% of all lung cancer diagnoses [1]. Stage at diagnosis is a key determinant of prognosis, and approximately 57% of patients present with stage IV disease [2]. Historically, advanced NSCLC was treated with systemic chemotherapy with platinum-based chemotherapy representing the standard of care. However, platinum-containing regimens are associated with significant toxicities, including nausea, vomiting, nephrotoxicity, ototoxicity, neurotoxicity, and myelosupression [3].

The emergence of immuno-oncology (IO) therapies has resulted in a paradigm shift in the treatment of advanced NSCLC. IO targets immune pathways allowing the body to recognize cancer cells as foreign and attack them (e.g., immune checkpoint inhibitors) or enhance overall immune functioning without targeting specific cancer cells (e.g., interleukins, interferons). When administered as monotherapy for treating advanced NSCLC, IO has been associated with response rates of approximately 20% in previously treated patients [4–8] and more than 25% in those who are treatment naive [9–12]. Compared with conventional chemotherapy, IO has been shown to have a comparatively benign safety profile, though the management of immune-related adverse events, which are frequently grades 1–2 but can affect multiple organ systems, is a prevalent concern [13, 14].

While IO has been labeled a “game changer” and “miracle in the making” [15, 16], there are many unanswered questions regarding its use in treating NCSLC [13, 17]. Among these, the patient-relevant benefits of IO have not been adequately delineated to date. Clinical investigations, including trials of nivolumab for the treatment of advanced squamous or non-squamous NSCLC, have shown IO to be associated with improvements in patient-reported outcomes (PROs). However, little is known about the long-term experiences of patients treated with IO, particularly those who may have survived more than a year after initiating treatment. The PROs recorded in clinical trials reflect patient experiences while on treatment (i.e., are germane to responders) and are limited by the content of the questionnaires used to assess outcomes.

The PRO measures conventionally used in studies of treatments for NSCLC were developed prior to the advent of IO and focus primarily on the severity and impacts of symptoms, both disease- and treatment-related, as well as functional status or capacity [18–26]. While these concepts are important, the patient experience of treatment encompasses much more. Health has been defined as “a state of complete physical, mental and social well-being and not merely the absence of disease” [27]. It follows that assessments of the effects of treatments on disease, including NSCLC, should include not only their impacts on symptoms and functioning but also the well-being achieved due to maintained or improved quality of life (QoL).

The concept of QoL emerged in the second half of the twentieth century as a way to “mediate between ideas of social progress and those of social and moral crisis” [28]. As medical-technological advances in the 1950s and 1960s increased cancer survival, there was a concurrent growth in interest in measuring QoL to ascertain whether interventions were doing more benefit than harm [28]. Cancer treatments that placed a heavy emotional and physical burden on patients—namely, chemotherapy—brought further attention to QoL and questions of whether a life on treatment was worthwhile [29, 30]. No single shared definition of QoL exists, though one generalization would be an individual’s evaluation of his or her life in the context of goals, expectations, and other reference standards, as influenced by values, and affected by health [27].

As IO extends the lives of patients treated for advanced NSCLC, the importance of assessing their QoL cannot be understated. Developing a more comprehensive understanding of patients’ experiences is critical to inferring the value of treatment. While receiving IO, a patient can be free of symptoms of disease progression as well as burdensome treatment-related symptoms, but without knowing his or her ability to engage in activities deemed important, social interactions and supports, and expectations for the future, any understanding of his or her QoL is incomplete. Moreover, for those patients who survive beyond IO therapy, it is important to understand not only the impact of late effects of treatment but also the individual’s ability to live a life that meets expectations, is free of disease-related stigma, and in which social integration is attained [31–43]. The limited knowledge that exists on IO and barriers to accessing this information may also cause patients consternation and distress. Knowledge gaps in regard to the profiles of likely responders and probable duration of treatment benefit may impact the emotional state of patients and accordingly their QoL.

Given the aforementioned challenges, an ethnographic study was conducted to develop a more comprehensive picture of the QoL of patients treated with IO for advanced NSCLC. Ethnographic research is a qualitative methodology with the aim to better understand people—their values, decision-making processes, and needs—by observing them in the context of their day-to-day lives rather than a controlled setting. Instead of drawing on existing measures of QoL, open-ended inquiry was used to facilitate an understanding of patients’ experiences during and after IO treatment and to identify personally significant issues pertinent to their QoL.

## Methods

### Approach

The study used a range of rapid ethnography techniques. Rapid ethnography utilizes multidisciplinary research teams and elicitation methods designed to maximize the amount of information obtained in a much shorter period of fieldwork than is typically allotted in “traditional” anthropological studies conducted for a minimum of a year to decades [44–47]. This methodology was developed in the context of rural development projects and public health programs where information on themes, needs, and key decision-making factors were gathered through the strategic and targeted use of ethnographic methodologies as opposed to large-scale surveys or extended anthropological inquiry [48].

Our team consisted of four research staff members with varying backgrounds who all had experience conducting ethnographic research. Participants were recruited through patient networks and cancer institutions and included consenting adult (age ≥ 18 years) patients treated with IO for stage IV NSCLC in Denmark, the UK, and the USA. Quota sampling was used to achieve comparable numbers of patients treated with IO for less than 1 year and 1 year or more. Data were gathered though the use of multiple written exercises, an observation guide, and in-depth interviews. Participants were given pseudonyms to provide for anonymity when describing their experiences.

To structure our research, which examined the experience of disease and treatment in context, we drew upon the social theory of illness as a major biographical disruption [49]. In studying the impacts of a chronic disease like cancer, which transforms a variety of major life aspects (e.g., day-to-day experiences, relationships, experiences of health, self-conceptions), we were able to better understand the underlying personal mechanisms and heuristics that constitute a life’s meaning. By examining the biographical disruption of living with NSCLC during or following IO treatment, we sought to identify the most personally important transformations in each participant’s life that might impact his or her self-definition of QoL.

**Fig. 1:**
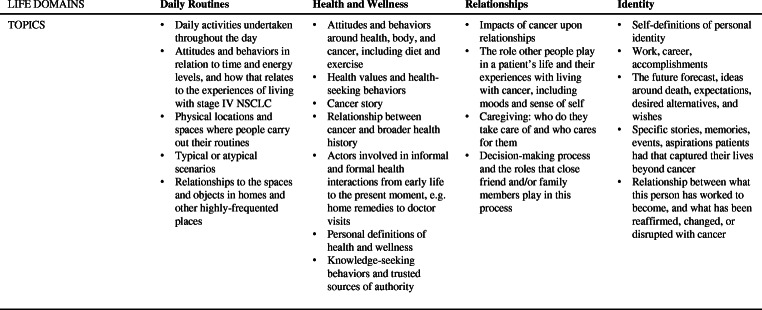
Selected life domains

In our fieldwork, we explored four life domains to analyze illness narratives: daily routines, health and wellness, relationships, and identity (Fig. 1). These four life domains are considered generative medical anthropological concepts of study and were selected to explore how patients’ lives were impacted by cancer and treatment [50–52]. For instance, within each of these life domains, we named topics to cover during our time with participants in their homes and while accompanying them to their hospital visits or wellness-related outings, such as yoga classes. Researchers did not have a linear script that they followed but rather a shared understanding of the key questions to guide participant observations. Our selected life domains ensured that data were solicited on the same topics across all participants.

### Study population

Out of 35 patients contacted for participation in the study, 24 met all inclusion criteria and provided informed consent. All recruited patients were diagnosed with stage IV NSCLC and had been on or were receiving nivolumab, pembrolizumab, atezolizumab, or durvalumab. Patients were divided evenly across field sites in Denmark (in and around Copenhagen), the UK (near Manchester, Birmingham, and Kent), and the USA (Southern California and the New York Tristate). The three countries were selected to represent a diversity in levels of market maturity of IO, healthcare systems, and access to care. In Denmark, IO treatment was more readily available compared with the UK and USA, and state-subsidized healthcare was easily accessible. In the USA, access to IO treatment was uneven as individuals had to have health insurance or government assistance to access care. In the UK, IO treatment was administered largely in the context of clinical trials because it had not been shown to meet the National Institute for Health and Care Excellence’s standards for cost-effectiveness and, therefore, was not reimbursed. For each participant, we also recruited from the “social ecology” (e.g., family members, friends, work colleagues, yoga teachers, nurses, and doctors) that surrounded him or her. Given our ethnographic approach, we sought to observe the social worlds in which patients lived in order to understand how cancer and IO treatment affected their relationships.

### Data collection

Fieldwork took place over a 4-week period with a central researcher assigned to each country. Each researcher spent 2 days in and around the patient’s home and accompanied the patient on a health-related trip (as defined by the participant). The latter ranged from tai chi classes to infusion treatments. Researchers conducted semi-structured interviews [53] with participants—as well as their family members and friends—while shadowing participants as they went about their everyday life. They also created specific exercises to open up conversations rapidly, including mapping out patients’ cancer journeys, describing patients’ life histories, administering self-assessments on holistic health, and taking home tours. During in-person visits, researchers collected audio-recordings, photographs, and field notes to record what they directly observed or heard, including quotations from interviews. Over 400 h of interviews were collected and analyzed for this study.

### Data analysis

**Fig. 2:**
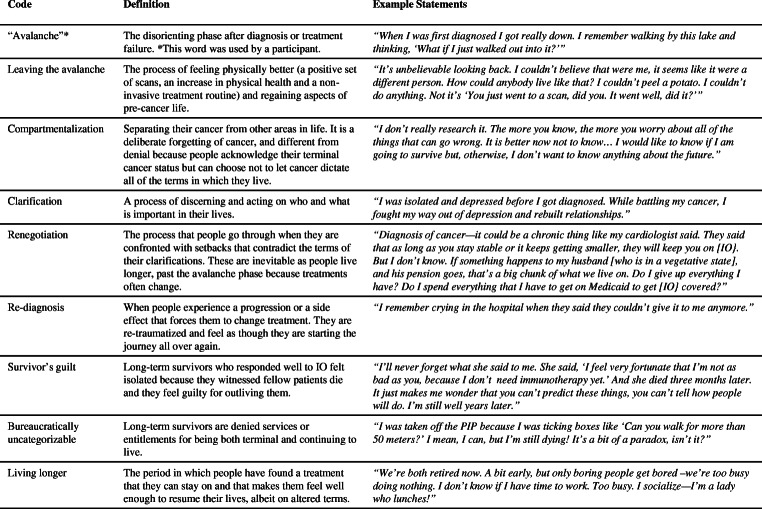
Creating codes for the corpus of data

Our data analysis process consisted of multiple methods derived from a grounded theory approach [54]. Researchers engaged in analytical notetaking, a common method in the school of symbolic and interpretive anthropology, which produces what anthropologist Clifford Geertz called “thick descriptions” of the behaviors of each participant to help unpack their cultural significance [55–58]. The process of creating thick descriptions—rich explanations of behaviors that help researchers decipher their contextual meaning—requires the analysis of data with an interpretive lens. Researchers applied methods of interpretative phenomenological analysis (IPA) [59] to raw data, where they identified codes in each set of thick descriptions across all notes. “Coding” is a way of indexing and organizing data in order to derive thematic ideas. In coding data, the researchers engaged in clustering exercises, creating codes to the corpus of data [60], which were used to generate grounded theory [61, 62] (Fig. 2).

## Results

Table 1 provides descriptive statistics for study participants. Among the 24 study participants, 15 were treated with pembrolizumab, 7 with nivolumab, 2 with atezolizumab, and 1 with durvalumab. Forty-six percent of patients were male, while 54% were female. The majority of participants (63%) were between 51 and 70 years of age, 29% were aged 71–90 years, and 8% were aged 31–50 years. Time since diagnosis ranged from 6 to 96 months, and time spent on IO treatment ranged from 1 to 34 months. Eleven participants had been on IO for 1 year or longer. Sixteen of the 24 participants were not experiencing tumor growth on or after stopping IO treatment. At the time of the research, 10 participants were receiving IO therapy, 5 had discontinued IO treatment, and 1 was receiving targeted therapy.

**Table 1:** Participant characteristics

Characteristic	*N*	Percentage (%)
Age group (years)		
31–50	2	8
51–70	15	63
71–90	7	29
Country		
UK	8	33
USA	8	33
Denmark	8	33
Gender		
Female	13	54
Male	11	46
Health status		
Stable^a^	16	66
Recent metastasis or secondary cancer	6	25
Awaiting results of first scan	2	8
IO treatment received^b^		
Pembrolizumab	15	60
Nivolumab	9	28
Atezolizumab	2	8
Durvalumab	1	4
Time since diagnosis		
< 1 year	3	13
≥1 year	21	87
Time on IO		
< 1 year	13	54
≥ 1 year	11	46

Tabled information was self-reported by study participants

^a^Participants classified as “stable” had no new metastases, no change in cancer stage, no adverse reactions to treatment, and no planned change in treatment

^b^One participant had experience with three IO therapies

Our study identified four predominant themes, including (1) the circular patient journey, (2) limbo, (3) community, and (4) renegotiation. In the following sections, we first discuss the cancer journey and the role IO plays in delineating a patient’s place within it. We then discuss the remaining themes, which relate to the second phase of the journey and patients’ experience of QoL. We address how patients who have been treated with IO exist in a kind of “limbo state.” Next, we discuss how participants exhibited a preference for peer sources of information about their cancer and treatments to help reduce uncertainties around being on IO treatment. Finally, we discuss the ways that participants found themselves engaged in a series of “renegotiations” with their identity, relationships, finances, and work.

## The circular patient journey

### Phase 1: “the avalanche”

We observed patients who were not cured of their cancer but living past their prognosis with the help of IO speaking about their experiences in terms of two general phases. The first phase is the “the avalanche,” a term we borrowed from Vladimir (47, USA) who was diagnosed in January 2017. For patients, the avalanche can be classified as a period of intense frustration and disorientation as they, their caretakers, and their healthcare providers (HCPs) search for an effective treatment following diagnosis. Vladimir described the phase—soon after diagnosis and when his treating physician recommended chemotherapy for a year—as a time during which he had “no energy and was nauseous all of the time.” As Vladimir explained:

“The period between my first doctor appointment and the diagnosis is a kind of avalanche of events, with lots of questions and tests being thrown at you. When I was told my diagnosis, it was a shock. It was an avalanche. It was a very depressive moment in my life.” [Vladimir, 47, USA]

**Fig. 3:**
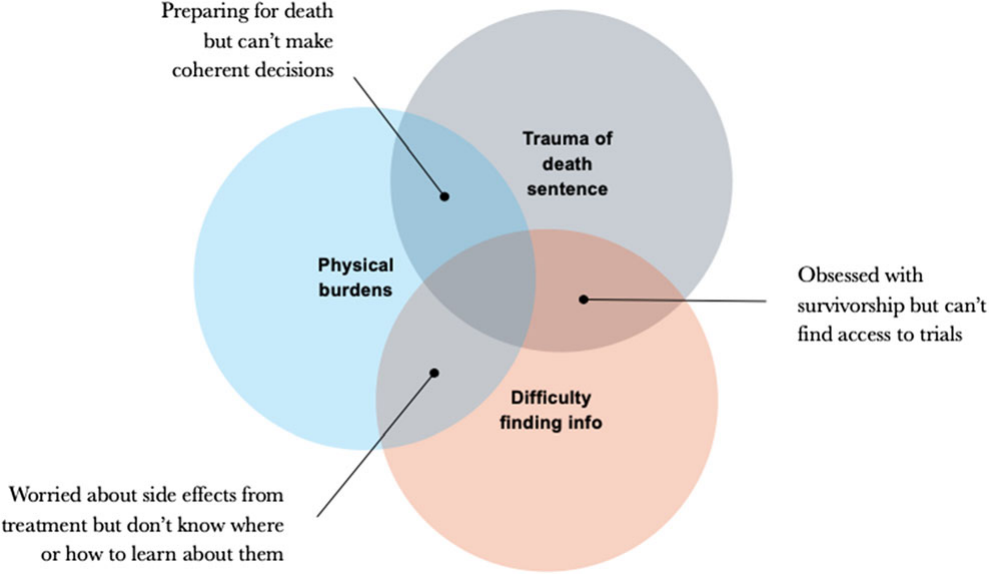
Overlapping physical, emotional, and mental states in which patients are making decisions, accessing care, and learning about their cancer

Vladimir’s quote captures what other patients tried to convey to us about the period following their diagnosis: everything that once made up their lives came crashing down around them like an avalanche. During this phase, patients experienced the trauma of diagnosis, intense physical effects of treatment, and a confused, overwhelming search for information about their condition (Fig. 3). The simultaneous occurrence of these challenges meant that patients not only had to face the new realities of their condition—making treatment assessments, managing side effects, and getting affairs in order—but did so with limited emotional and physical capabilities.

Jodie (59, UK) described an experience analogous to the avalanche phase when recalling her first diagnosis in June of 2016, when she was told she had about a year left to live. Jodie was obsessed with surviving for the sake of her children and grandchildren but did not know how to access the latest treatments in the UK. She reported having “near-comatose” pain for 3 months and being shocked by the loss of her role as the caregiver of her nine grandchildren. Overwhelmed by the task of getting into a trial—the only way to access IO in the UK at the time—Jodie like others relied on her physician to guide her through the process. Jodie’s husband recalled, “We more or less left it all in the hands of the cancer center, and we had never even heard of that medication.” Patients like Jodie remembered being asked about their goals for treatment and given a menu of options. However, they deferred to their doctors because they lacked a frame of reference to make decisions about their treatment.

The avalanche was also described as a time when cancer had taken over everything in the patient’s life. Patients reported staying in their homes and feeling too weak to engage in everyday activities, jobs, and family roles. One patient, Judit (65, DK), who was diagnosed in December 2017 and had received both radiation and IO, initially felt as though her cancer was a “death sentence” and that her “life was over.” She described the first few months following her diagnosis as both “physically and mentally terrible.” Judit was told there was a fifty-fifty chance she would survive through the end of the year and commented, “When they first told me, it felt like I was already dead. I thought I was going to die right away.” As was the case for many patients, Judit was overwhelmed and unable to focus on the people, places, and activities that had defined her life prior to diagnosis.

### Moving beyond the avalanche

For many patients, movement beyond the avalanche occurred when they experienced a series of positive scans, felt a marked sense of improvement in their physical health, and were able to stay on a routine treatment schedule. While movement beyond the avalanche was not unique to patients who responded to IO, those who did respond to IO after failing to respond to standard of care treatments were more likely to experience this transition. The transition was not immediate, and patients did not discuss going through a transition explicitly. Instead they spoke about phases in which they were very sick, not able to get out of bed, and receiving hospice care, and then other phases in which they were able to resume their lives, felt healthier, and found a treatment that was either preventing their cancer from growing or shrinking tumors. Participants often reflected on times when they were very ill as if they could not believe their health had changed so drastically. For example, Jodie (59, UK) thought it was remarkable that she went from being a patient unable to perform her role as the primary caregiver and homemaker to resuming that role once again. She commented, “It’s unbelievable looking back. I couldn’t believe that were me, it seems like a different person. How could anybody live like that? I couldn’t peel a potato. I couldn’t do anything.” Jodie’s husband joked, “I was cooking while she was doing poorly, and I think the prospect of that going on for much longer really made her get better.”

### Phase 2: “living longer”

The second phase that participants generally described when speaking about their experiences of cancer was “living longer,” a term that encompassed time on a steady treatment plan without progression or adverse events so severe that a patient needed to stop therapy. Those patients who had been in the living longer phase for years when researchers met with them described this phase as a time when they could resume parts of their pre-cancer lives and even “forget” about their cancer at times. In this phase, patients reported spending time outside the home, resuming household roles, returning to work, and engaging in the mundane routines of everyday life. We term this behavior “compartmentalization” due to patients’ ability to psychologically cordon off cancer from the rest of their lives. By intentionally delineating boundaries for when, where, and with whom they thought about and talked about their cancer, patients could acknowledge their disease without allowing it to dictate the terms of everyday life.

One participant, Ditte (60, DK), who was diagnosed in April 2017 and underwent chemotherapy before receiving IO in May 2017 and responding to treatment, explained that “cancer lives in the hospital.” Ditte kept her mind occupied with puzzles and television, hula hooped daily in her backyard for exercise, and enjoyed interacting with her dog, which had a seemingly endless amount of energy. While Ditte almost seemed surprised by the reality of her condition, and moments of reflection prompted by the interview were painful for her, she acknowledged that her participation in this study was a “great and special experience.” ﻿

Another example of someone who was able to engage in the everyday mundanities of life during the living longer phase was Mark (48, USA). Mark was a pediatrician in New York who had been living with stage IV NSCLC since 2011. Mark experienced the living longer phase while receiving an effective form of chemotherapy for 5 years between 2012 and 2017. Due to his continued ability to work and fulfill fatherly duties during this time, Mark and his wife decided not to disclose his diagnosis to their two sons. Moreover, Mark continued to uphold his reputation among his colleagues as the hardest working pediatrician in the emergency room and kept his preferred long hours spending time with his young patients and their parents.

As demonstrated in the above examples, patients reported a higher QoL in the living longer phase due in large part to their ability to engage in everyday activities found to be personally meaningful. Those who were receiving IO treatment after prior treatment with chemotherapy reported improved QoL due not only to the experience of fewer adverse events but the impact of those events on their ability to engage in activities that defined a “normal” life.

### Cycling between phases

For many patients, the journey was not a linear one of consistent improvement or decline but rather a circular one in which they returned to the avalanche phase when they had a progression or treatment setback, such as an adverse event that required the cessation of treatment. To paraphrase several participants, this experience of cycling back to the avalanche was “like going back to square one.” Each time a person re-experienced the avalanche, he or she carried the emotional and physical baggage of everything that had gone on before.

As participants cycled in and out of the avalanche and living longer phases, they coped with variations in their ability to compartmentalize cancer. For example, after five stable years on chemotherapy, Mark (48, USA) suffered a sudden stroke and was told that his cancer had spread to his lymph nodes. He described this experience as “being diagnosed all over again.” As Mark’s physical health declined, he could no longer play catch with his sons and had trouble listening to them at the dinner table due to pain and nausea. This compelled Mark to disclose his diagnosis to his children, which impacted his sense of empowerment and control. Mark also found it increasingly difficult to hide his oxygen tank and other medical paraphernalia in the house as he needed to use these items more frequently. Mark’s negative experience suggests that continued inhabitance of the living longer phase is linked to a patient’s ability to compartmentalize his or her disease.

## Limbo

To receive a life-extending treatment—as IO was perceived by many of our study participants—is a positive experience. Twenty of the 24 participants were elated that IO had helped them for at least a period of time arrest the progress of their disease, and 16 participants were in a stable condition at the time of our research. However, prolonged survival created new challenges of which the foremost was coming to understand one’s state of health. Patients who responded to IO had their disease progression arrested, and many had such minimal side effects that they were able to return to lives that felt “normal.” At the same time, these individuals knew that their treatment could stop working at any moment, or that life-threatening side effects could require them to discontinue treatment, returning them instantly to their prior status as “terminal” cancer patients. For this reason, several participants described themselves as being in a kind of “limbo” state. They were sufficiently well to be working, raising their children, and engaged in leisure activities, but they were not cured, and their futures were uncertain.

The situation of Alice (62, UK) represented the worst fears of many patients. IO had carried Alice through her illness for a little over a year and had enabled her to retain her role as the headstrong and resilient matriarch of her family. “It worked really well,” recalled Alice’s daughter, “You could still do everything.” However, Alice eventually developed a severe itch that required her to stop IO treatment, and her physician placed her on chemotherapy. In a disappointing turn, on the day of our visit with Alice, her doctor broke the news that her chemotherapy was not working and was causing renal damage. Alice commented, “Everything’s gone off since I’ve changed from my trial [IO] to this chemo.”

The insecurity of the limbo state affected a range of decision-making activities, from daily diet to vacation planning. Several participants followed self-imposed rules in an effort to demonstrate agency over their cancer, which suggested an awareness of their own mortality. For example, Jodie (59, UK) expressed a fear of traveling ever since she had experienced a severe side effect while on holiday and created a self-imposed travel ban. When we met with her, Jodie described how her doctor finally convinced her to take a much-needed vacation.

“I didn’t go on any trips for a long time, but then I asked my doctor about flying and how it could affect my lungs, and she told me: ‘Why do you think we’re making you better? So you can go on holidays!’ I see her point, so I told my husband to book a trip right away. But I still won’t travel over four hours.” [Jodie, 59, UK]

Despite acquiescing to her physician’s recommendation, Jodie still had self-imposed rules on where and how far she would travel that correlated with her experiences and expectations for a life with terminal cancer. For instance, Jodie would not take trips that were more than 4 h in length in case she needed to return home quickly to see her doctor. She limited her travel to flat and cool places after a trip to Portugal left her more fatigued than usual. In addition, Jodie would only travel to places with a perceived good hospital system in case she needed medical attention. By imposing these restrictions, Jodie was able to travel with her family, which she equated with having a higher QoL, while also attending to her concern over her ongoing and uncertain condition.

For many patients, the limbo state also affected daily routines. Vladimir (47, USA), who cycled on and off of chemotherapy for a year before receiving IO, wavered back and forth between indulging in rich food and exercising more self-restraint in order to support a potentially longer lifespan. Much of our interaction with Vladimir centered on his preoccupation with how the things he enjoyed were also the things that were “bad” for him. This included food, exercise, doctors, and recreational activities. Vladimir commented on people he knew who got sick in spite of obsessing over their health, while others did nothing special for their health and lived long lives. While Vladimir experienced generally good QoL during his treatment with IO, he was torn between doing everything in his power to increase his odds of survival and living life as if he did not have cancer. At the time we met with him, Vladimir was caught in a state of limbo in which he was preoccupied with potential future consequences of his current health behaviors even though he had been maintained successfully on IO for many months.

In addition to feeling “in limbo” in their personal lives, some participants felt institutionally “in limbo.” Although they may have been entitled to cancer support center services—including free classes for yoga or cooking, support groups for family members, and help in navigating the healthcare system—most study participants declined to utilize these services because they did not feel “sick enough” to “deserve” them. For instance, Jodie (59, UK) refused to utilize services provided by Maggie’s Centres because she thought they should be reserved for “traditional, sicker people who need it.” Even when a researcher asked Jodie to accompany him to a Maggie’s Centre for a visit, she did not feel it was a place for a patient like her. She perceived her QoL to be higher than that of peers who were not responding to treatment and expressed discomfort using resources that were more appropriately directed to them.

Recognizing how rare their cases were in the wider landscape of lung cancer care, participants also felt uncomfortable sharing their stories with cancer support centers or groups. Steven (54, UK), a retired merchant seaman, was diagnosed in 2014 and initiated on chemotherapy. After his chemotherapy failed, Steven was placed into hospice care, and as a last resort, his physician started treatment with IO. This had what Steven called a “Lazarus effect,” improving his health dramatically such that he was able to resume golfing and other activities that were previously beyond his capability. Steven was a vivid, compelling speaker, which made it understandable why support groups frequently requested him as a presenter. However, Steven routinely declined, saying that “Advocates ask me to come in and share my miracle story, but I say no. It seems a bit selfish*…*I don’t want to be around sick people when I’m feeling good.” Steven confided that he did not feel he was completely “out of the woods,” and being superstitious he was reluctant to beckon misfortune by sharing his story.

This “institutional limbo” is challenging because the patients treated with IO who enjoy some semblance of QoL still require support with, and help advocating around, their unique and half-understood situations. For instance, existing policies pertaining to patients with advanced cancer often do not account for them living long with their terminal disease. In our study, some of the longer-living participants were rejected for services and entitlements because they outlived prognoses or were maintaining good health. One such patient, Dana (52, UK), was a former accountant with the National Health Service who had two sons. Following her diagnosis in 2015, Dana applied for and began receiving disability payments with special allowances for the terminally ill. However, after 3 years of receiving these payments, Dana’s renewal request was rejected because she was no longer deemed to be “end-of-life.” Dana’s benefits were only continued after her healthcare provider intervened on her behalf. She explained, “I was taken off the [disability service] because I was ticking boxes like ‘Can you walk for more than 50 meters?’ I mean, I can, but I’m still dying! It’s a bit of a paradox, isn’t it?” Dana’s comments draw attention to the fact that traditional measures of functional status applied to patients with terminal illness do not apply to her because she was able to resume her normal activities. However, Dana still lived with the possibility of succumbing to her cancer, and the continued receipt of disability payments allowed her plan for the care and well-being of her sons after her passing.

The lack of institutional support also extended to patients who were considered too much of a liability despite having minimal symptomatology. Before Ditte (60, DK) was diagnosed, she and her husband spent most of their vacations traveling abroad, including trips to Thailand and South Africa. Yet despite being stabilized on IO after failing chemotherapy, Ditte no longer qualified for travel insurance. Danish insurers would not cover someone with a terminal cancer diagnosis without the permission of their treating physician. Travel insurance was considered essential by Ditte and her husband because without it the Danish healthcare system would not cover treatment costs in the event of a medical emergency. Reluctantly, Ditte resigned herself to domestic trips, though her greatest hope was to travel to the south of France, where her family had been visiting yearly for decades.

### Community

Our study participants were receiving IO at a time when standard knowledge and experience with this class of treatments were limited across the medical community. Most participants reported receiving less detailed and clear information about side effects than had been received for treatments like radiation and chemotherapy. For instance, Karl (71, DK) struggled to get the information he needed from his HCPs. Despite being a member of every Danish patient organization he was eligible to join, Karl could not find anyone with whom to discuss and share practical advice around IO. “The patient advocacy events are very general,” he said, implying that they cater to a general population of cancer survivors. “I want someone I can talk specifically about IO with. But it’s so new that the nurses don’t really know everything, and the doctors are very careful not to say too much.”

Lacking sufficient guidance from the medical establishment, patients instead turned to peer communities online to get reassurance and read self-reported experiences on IO. While this behavior is not uncommon among cancer patients undergoing more established treatments, it was notable how widespread it was in our sample. Our participants reported a nearly unanimous desire for peer testimonies when learning about IO, and 12 participants joined online communities, including Facebook and Google Groups, where they could pose questions and solicit advice from others treated with IO.

Much of the discourse online revolved around side effects. Dave (66, USA), a former salesman in the print business who was diagnosed in 2006, considered himself a kind of crowdsourcer of side effect data online. “I moderate an IO group, we have maybe a thousand people all over the world…We’ll learn about side effects before they’re even published. We were talking about thyroid issues before it was a common issue.” Some patients used the Internet to validate their beliefs that the symptoms they experienced were treatment related. In one instance, Whitney (59, UK) found that she gained weight while treated with IO. Her doctor simply attributed the weight gain to “aging.” However online, Whitney found patients of various ages who experienced weight gain while treated with IO, which supported her belief that her weight gain was due to her treatment.

With the help of their peers, many patients also sought to evaluate whether the symptoms they experienced should be a cause for concern. Patients who were maintained on IO frequently communicated with online communities of laypersons to ascertain whether a side effect they experienced was troublesome. A prevalent concern among patients was that any frank communication about symptoms with their treating physician could result in discontinuation of IO treatment. Patients demonstrated a lack of awareness that their HCPs could assist them in addressing side effects, which could potentially enable them to stay on IO therapy longer.

Participants also turned to IO forums to learn what their lives might be like after IO treatment, particularly in the scenario where IO might cease working for them. This was especially pronounced in cases where patients had recently undergone a “progression,” such as a growth in their tumor, even if that growth was not yet deemed threatening to their health. Said Dave (66), the US-based former businessman, who had survived stage IV NSCLC for 12 years:

“When you’ve been stable that many years on the same drug you sort of get complacent. But now I’ve had three progressions in the last few years. It’s no longer a question I know I’m going to have progressions at some point, and I need to have treatment options lined up…I kept thinking you know it’s like playing Russian roulette, one of these times I’m going to spin the chamber and it’s going to go off.” [Dave, 66, USA]

Many patients expressed a preference for using IO forums to discuss comorbidities. Often, there were questions about how IO treatment might intersect with or affect treatments for comorbid conditions, such as heart disease. Patients noted situations in which online communities were able to provide information when their HCPs were unable to do so. For instance, Dave had an enlarged heart, a condition that predated his cancer, and feared that no one would be able to share information about how IO impacted this condition. Using online resources, Dave was able to identify other patients who had had similar experiences.

Overall, online peer communities provided patients with practical information that helped them reduce anxieties or unknowns about what life with IO treatment might be like for them and their families. Having access to information about potential trials, interpreting side effects, and learning about how other patients dealt with comorbidities helped participants maintain their QoL because they could apply this information to decisions around their own health. These online resources were not used to gain emotional support but rather access to what was perceived as credible “on-the-ground” information.

### Renegotiation

Prior to the availability of IO, there was a “set playbook” for patients with terminal lung cancer that predicted a certain number of months to live. The tasks ahead were not easy, but they were more or less understood. Patients confronted their mortality, planned their estates, and spent time with loved ones. However, among the patients we interviewed, most of whom had received a terminal diagnosis, the sudden and dramatic impact of IO rewrote that “playbook.” While the favorable response to IO that most patients experienced was a blessing, it also introduced complications. These individuals had prepared for their deaths and were suddenly given years to live. They were exposed to a kind of psychological whiplash, and as a result many reported having to “renegotiate” the terms on which they were living.

With the relative wellness brought on by IO treatment, many participants renegotiated roles with family members and partners, often reclaiming a degree of autonomy that they thought had been ceded for good. Many patients went out of their way to unburden their family members from their cancer. For example, Alice (72, UK) elected to take a 3-h bus ride to her IO infusion every 3 weeks because she did not want to ask her son or daughter to take time off from work to drive her to her appointment. Alice said, “They have their own lives, you know? Work and taking care of your daughters doesn’t just hold on and wait when I need to go to my appointments. So, I don’t ask much of [my daughter and son].”

In more dramatic instances, entire relationships had to be renegotiated, sometimes in prolonged and difficult ways. Søren (60, DK) was by his own admission an alcoholic and “terrible father” to his two daughters from whom he had become estranged in his 40s and 50s. When he received a terminal diagnosis, Søren told his daughters how little time he had left, and they tentatively reconciled. Not long after, Søren was admitted into hospice. However, Søren was subsequently placed on IO therapy and, seven years later, has been thriving ever since. His extended survival has introduced a sustained, tense dynamic with his daughters who in a sense “didn’t sign up for this.” They were prepared to grant their father absolution and say goodbye but now field calls from their father twice per day. Søren’s relationship with his eldest daughter remains particularly tense. During a visit to Søren’s home, she told the researcher, “Sometimes I think it would have been a relief if he didn’t make it.”

Researchers encountered a similar tension in the marriage of Peter (67, DK) and his wife Tine. Tine had retired early in order to take care of Peter following his diagnosis in 2013 and when surgery, radiation, and chemotherapy failed to halt the progression of his cancer. Recalling that period, Peter noted, “In the end, they gave up on me. They said there was nothing more they could do.” However, when describing his current health following an almost miraculous recovery during experimental treatment with IO, Peter said, “I mark my health score 99 just to make room for improvement.” Upon meeting Peter and Tine, Tine disclosed her regret for retiring early, and she seemed slightly annoyed with Peter’s carefree nature, including his tinkering joyfully around the home, playing bingo, and maintaining an aviary. The gusto with which Peter now lived his life (exhibited in his statement, “You never know the day before the pub’s going to close,” quoting a Danish saying similar to carpe diem) seemed incongruous with his wife’s disappointment in regard to her career.

Financial decisions also had to be renegotiated. A number of patients retired or quit work based on the amount of time they estimated they had left. Those whose survival had outstripped expectations were often compelled to re-examine their financial situation. Joe (72, UK) retired early from carpentry as soon as he was diagnosed in 2015. After surgery and chemotherapy failed to cure him, he was initiated on IO therapy and has been kept on this treatment ever since. Joe now finds his lack of employment difficult because he has lost his sense of purpose and income. On the day we met Joe, he was mulling over job orders to cover financial needs during his retirement, which now looks to be longer than expected.

Renegotiating work was also central to the IO experience of Whitney (59, UK), an energetic older woman with a quick wit. When Whitney was initially given a terminal diagnosis and placed on chemotherapy, she quit her job as a financial services compliance officer. For Whitney, her diagnosis was a clarification that her job provided little meaning in her life and was simply a means of financial support. Following a second round of chemotherapy and subsequent IO treatment, Whitney’s health improved dramatically. She recalled, “I knew I had to start working full-time again. My salary had gone down, but not my mortgage. Not my phone bill.” Whitney elected to return to her job because she felt unable to land other employment as an older worker. Although she has had to contend with a variety of adverse events, Whitney conveyed a belief that her employer has not given her adequate leeway to deal with the impacts of these events. For instance, when Whitney would tell her boss and coworkers that she was tired and needed a rest, she sensed that they saw her as being “lazy.” Whitney described the experience of renegotiating her career since starting IO treatment as very emotionally taxing.

A final way in which patients renegotiated their relationship with their cancer involved decisions as to where and when they “allowed” it to be discussed. While many patients found it impossible to keep “cancer talk” out of the home while receiving all-consuming and highly visible treatments like chemotherapy, those treated with IO more commonly found they were able to “compartmentalize” their cancer and any discussions of it. For Barb (52, USA), the ability to keep discussion of her cancer out of the home led to a feeling of having her life “normalized.” While on IO, Barb avoided speaking about her cancer with her husband and two children in order to protect them from the emotional burden of her disease, which she remembered all too well from her time on chemotherapy. According to Barb, “[My son] would be like, ‘You okay? You alright? You okay?’ ... He goes into high alert and gets stressed out really easily… And my husband, he, I don’t want to overwhelm him. And my daughter, I just want her to enjoy being a graduate.”

## Discussion

This research is intended to add a personal and contextual lens to a growing body of work examining the experiences of patients treated with IO for stage IV NSCLC and the sufficiency of existing PRO—including QoL—measures for these patients [63]. Our study employed a mixed-methodological approach, drawing from both grounded theory and interpretative phenomenological analysis. QoL is not something that participants spoke directly to or defined explicitly. Instead, patients spoke about how they were living. The main findings of our study are based in human stories that showcase the paradoxical existence that patients living with advanced NSCLC during or following IO can experience, which has implications for the definition and measurement of QoL.

The first set of findings outlined the two major phases of the patient journey and the process by which patients regain some sense of normalcy and stability after the trauma of diagnosis and treatment initiation. One of the distinguishing features of the living longer phase was participants’ ability to compartmentalize their cancer. The discussion of compartmentalization—where patients deliberately delineate boundaries for when, where, and with whom they think about cancer—should not be confused with existing theories around denial [64, 65]. Denial has been studied as an element that impacts patient QoL and is defined as a general or adaptive coping mechanism developed to avoid confrontation with the full consequences of disease and treatment. Compartmentalization is not the rejection of the reality of disease but instead the ability to deal with and acknowledge cancer in specific ways. The relationship of compartmentalization to QoL should be further delineated in future research.

Though existing research suggests that the experience and conception of QoL evolve throughout the treatment journey [66], few conceptualizations of that journey reflect the circular nature of what our participants described. This is perhaps in part due to the fact that longitudinal research on patients with NSCLC is lacking, especially as life expectancy with this disease has historically been short. This discrepancy suggests that the experiences of those living with NSCLC during or following IO treatment deserve further investigation in order to better understand their relationship to the patient journey and QoL.

The second set of findings around the theme of living in limbo underscores the liminal status that participants felt and the strategies they adopted to address their situations. Most participants navigated a sense of “in-between-ness;” that is, they were technically “terminal” though feeling well enough to live day to day. Participants were not confused over what “terminal cancer” meant but rather were experiencing a second lease on life after receiving a terminal diagnosis. Patients treated with IO for stage IV NSCLC may be underserved by existing support systems due to explicit exclusion or feelings of alienation. In outliving the timeline of a terminal diagnosis, patients were bureaucratically uncategorizable, where they lost access to benefits because they did not fit under any existing categories for patients with a terminal illness. This may have been due to a lack of awareness about the potential for longer-term survivorship in NSCLC while receiving IO as well as a lack of communication between patient support resources and the medical community. In addition to this external barrier to support, we also observed that over time patients developed an internal conception of themselves as outsiders in the broader lung cancer community, where they felt undeserving of typical resources. The isolation of long-term survivors is distinct from stigmatization in which an individual “experiences devaluation by others and exclusion from social relationships” and generally reports lower scores for “behavioral and interactional dimensions of QoL” [67]. In our research, we observed survivors isolated themselves because they were hesitant to complain about their own circumstances as they outlived others or lived comparatively healthy lives. Rather than lower self-reported QoL as a result of stigma, self-censorship may lead to inflated perceptions of QoL as patients relationally diminish their struggles. Whether due to external or internal factors, the inability of patients to access certain benefits has important implications for the assessment of QoL that warrant further inquiry.

The third set of findings around community suggests that patients are burdened by the lack of established and practical information on IO from HCPs. Instead, they look to peer communities, especially those online, to supplement their knowledge base and aid their decision-making. This felt need for “on-the-ground” information about treatment paradoxically increased as patients remained stable on IO. Our observations suggest three reasons for this. The first is that the longer a patient lives, and chances for continued survival decrease, the greater is the felt need to identify additional treatment options. Patients want a “plan B” because they anticipate an eventual change in events. The second is that the longer a patient remains on IO the more likely it is that he or she will experience side effects that may not be commonly acknowledged in the medical literature. Patients in this study felt that the nurses and doctors they interacted with were hesitant to draw explicit connections between their side effects and IO because of the lack of established research around this new class of treatments. The third is that as patients live beyond their original prognosis, they are able to turn their attention to other health concerns, and there is limited research and standardized practices around treatment for health issues concomitant to stage IV NSCLC. Recent research finds that a significant percentage of patients treated for NSCLC experience decisional conflict around their therapy and feel uninformed [68]. While our research corroborates findings of patients feeling uniformed and conflicted, we observed patients to experience significant emotional strain as they continued living in limbo while seeking information on their health and treatment. As such, we believe this theme of information-seeking should be re-evaluated in relation to the duration of survival. Peer information was a strategy employed for when people were seeking to maintain QoL while living in a limbo state, helping them to find direction and make decisions about how to age, plan family life, and attend to other non-cancer-related health issues.

The fourth set of findings centers on the theme of renegotiation in which patients rethink their priorities or revise their post-diagnosis plans once they realize that their lifestyle changes are not sustainable. Renegotiation reflects an ongoing shift between patients planning for the end of life and being confronted with continued survival. We observed patients face challenges in sustaining priorities and behaviors over time after having made lifestyle changes with the assumption that their life expectancy would be limited. Our findings suggest that patient experiences and definitions of QoL evolve in relation to this process and individuals’ ability to successfully clarify and renegotiate as needed. The interpretation of this process should stand in contrast to the correlation between attainment of “personal goals” and QoL, which has been researched in several contexts [69, 70]. Meeting achievable life goals (e.g., attending a wedding of a loved one, getting a new promotion) has been linked to higher QoL in that it is associated with a recapture of personal identity, a feeling of well-being after recovery, and a sense of normality in life [71]. However, rather than a focus on attaining finite goals, we observed an ongoing evolution of what mattered most, which suggests a need to reconsider how to measure this aspect of QoL. Patients found it necessary to revise decisions previously made in order to continue living a normal life and maintain everyday relationships with those around them.

## Limitations and suggestions for further research

This research was conducted at a particular time in which participants were experiencing their cancer journeys and relied upon their recollection of events. More research based on repeated interactions with patients to track them in real time could deepen our understanding around their decision-making, the challenges they face at different moments in the journeys, and their interpretation of the information given to them at physician visits. Requiring a long period of involvement in research could present a challenge in terms of recruitment as prospective participants might prefer to spend time with family members and friends rather than with researchers. However, if this research could be performed, then we would gain a more concrete perspective on how patients’ lives change over time and how their changing baseline affects evaluations of their life and treatment.

It is also possible that these research findings are not applicable to all cancer types or even other stages of NSCLC. Given the low survival rate of patients with stage IV NSCLC treated with conventional therapies, the benefits of IO could be particularly significant for this group. Therefore, it is difficult to suggest that the findings based on this sample as a whole are universally applicable to other cancers. More research is needed on the impact of IO on long-term survivors of other types of cancers as well as NSCLC stages.

## Conclusion

Our findings suggest a number of implications for HCPs, patient advocacy and cancer support groups, and QoL researchers. HCPs might facilitate discussions with patients about the possibility of experiencing a life in limbo and seek to better understand the renegotiations accompanying IO treatment to comprehend how IO affects changes in patient decision-making. Support centers might create dedicated resources (e.g., support groups) for patients who have responded well to IO therapy. In order to educate themselves about the ways in which patients articulate their experiences on IO and be aware of potential sources of misinformation, HCPs and advocacy groups might familiarize themselves with the online spaces used by patients to gather information from their peers.

Last, QoL researchers might seek to identify the ways in which patients renegotiate and compromise on what matters most to them in order to manage uncertainty as well as the factors related to their ability to successfully separate cancer from other parts of their lives. Given the content of available PRO measures, this may suggest the need for development of new instruments.
